# Transfer-Efficient Face Routing Using the Planar Graphs of Neighbors in High Density WSNs

**DOI:** 10.3390/s17102402

**Published:** 2017-10-20

**Authors:** Eun-Seok Cho, Yongbin Yim, Sang-Ha Kim

**Affiliations:** 1 Department of Computer Engineering, Chungnam National University, Daejeon 301-747, Korea; escho@cnu.ac.kr (E.-S.C.); ybyim@cclab.cnu.ac.kr (Y.Y.); 2 Information Technology Management Division, Agency for Defense Development, Daejeon 305-150, Korea

**Keywords:** face routing, distributed processing, planar graph, transfer efficiency, high density WSN

## Abstract

Face routing has been adopted in wireless sensor networks (WSNs) where topological changes occur frequently or maintaining full network information is difficult. For message forwarding in networks, a planar graph is used to prevent looping, and because long edges are removed by planarization and the resulting planar graph is composed of short edges, and messages are forwarded along multiple nodes connected by them even though they can be forwarded directly. To solve this, face routing using information on all nodes within 2-hop range was adopted to forward messages directly to the farthest node within radio range. However, as the density of the nodes increases, network performance plunges because message transfer nodes receive and process increased node information. To deal with this problem, we propose a new face routing using the planar graphs of neighboring nodes to improve transfer efficiency. It forwards a message directly to the farthest neighbor and reduces loads and processing time by distributing network graph construction and planarization to the neighbors. It also decreases the amount of location information to be transmitted by sending information on the planar graph nodes rather than on all neighboring nodes. Simulation results show that it significantly improves transfer efficiency.

## 1. Introduction

A wireless sensor network (WSN) is a multi-hop wireless network composed of a number of randomly deployed sensors capable of communicating for a specific purpose. Due to their ability to sense information about the environment, process the sensed data, and deliver it to remote locations, various WSN applications have been continuously developed and researched [1,2,3,4]. In addition, diverse research on resource management and efficient routing to extend the lifetime of a WSN have been carried out because the sensors have small, limited resources and are often inaccessible after deployment [5,6].

For data delivery in WSNs, geographic routing has been considered as an appropriate routing strategy due to being stateless under the constrained resources and a number of data transferring methods using geographic information in WSNs have been studied [7,8,9,10]. Geographic routing using the location information of a node instead of its network address has been classified as single-path, multi-path, and flooding-based [11]. A single-path-based routing algorithm that performs routing with less computational complexity and fewer required resources are preferred because of the insufficient resources such as power and bandwidth at each node [11,12]. Greedy routing and face routing are used in the single-path approach.

Greedy routing forwards a message to the neighbor node closest to the destination, but routing fails if there is no neighbor node closer to the destination than the message transfer node. However, face routing transmits a message along the face boundary of a planar graph built by eliminating intersecting edges that can cause loops in a network graph. It is used as one of the methods for handling communications in a void area where a message cannot be forwarded by greedy routing [13]. In order to detour void areas, other routing protocols (e.g., ITGR [14], HDAR [15]) have been proposed to deliver messages along a path that is unaffected by holes after hole detection.

Face routing involves a planarization process that removes intersecting edges in a network graph in order to construct a planar graph, whereby the generated planar graph should not be segmented due to the eliminated edges. The Gabriel graph (GG) [16] and the relative neighborhood graph (RNG) [17] are representative planar graphs in which the network is not cut off when they are constructed using only local information [18]. In order to perform planarization while preventing the segmentation of a network, GG and RNG remove long edges that might be an intersecting edge in a network graph. Therefore, planarization results in a planar graph consisting of only short edges. However, this causes inefficiency because a message is sequentially transferred to the remote node within the radio range along the nodes on a face boundary made up of short edges even though it could be directly forwarded.

In order to mitigate the inefficiency, face routing using the location information on all nodes within 2-hop range has been proposed [19]. It forwards a message directly to the most remote neighbor located on the routing path within radio range. In this method, a message transfer node builds a local full network graph within radio range using 1- and 2-hop node information, performs planarization to construct a local full planar graph within radio range, discovers the most remote node to which a message can be forwarded, and sends a message directly to the selected node without traveling via intermediate nodes. Since the location information on all the 2-hop nodes as well as the immediate neighboring nodes must be transmitted to the message transfer node, the amount of information to be transmitted is increased compared to legacy face routing. In addition, because the method requires the construction of a network graph using the increased information and then performing planarization, the number of calculations is increased, as is the consumption of resources such as memory and energy.

This phenomenon makes the efficiency drop sharply due to the long computation time and the high energy consumption caused by the large number of nodes deployed within radio range and their position information in a high density WSN. In addition, whenever a node within 2-hop range is added or deleted and regardless of whether it affects the routing, the message transfer node always receives information on the changed node and performs network graph generation, planarization, and routing again.

In this paper, we propose a novel face routing method that uses the planar graphs of the message transfer node’s neighbors to forward a message to the most remote neighbor on the route within radio range without traveling via intermediate nodes to improve transfer efficiency and to solve the problems previously mentioned in existing face routing methods.

In the proposed face routing, a message transfer node receives the information on the planar graph of its neighbors and it constructs a local full planar graph within radio range. Using the generated planar graph, the most remote neighbor node on a routing path is discovered and the message is transferred directly to the node without traveling via intermediate nodes. Unlike face routing using 2-hop node information, the load for generating the local planar graph is distributed to each neighbor, which reduces the amount of location information to be transmitted by sending only the information on the planar graph nodes rather than on all of the neighboring nodes. In addition, the efficiency can be improved by virtue of only receiving information when a node changes and recalculating the routing if there is an influence on the planar graph rather than every time a node is added or destroyed. That is to say, if a node is added to the network but it is removed during the planarization process of the neighbor, the changed node information need not be transmitted to the message transfer node and it is not necessary to recalculate the routing. In addition, if a node is removed from the network but has already been deleted in the existing planar graph, the changed topology need not be transmitted. Furthermore, in order to improve transfer efficiency while balancing energy consumption of each node, the message transfer node, if its remaining power is low, sends the message to the nearest neighbor along the face boundary instead of forwarding it to the remote node to minimize energy consumption.

The rest of the paper is organized as follows. In Section 2, we provide a review of related work. In Section 3, we discuss the problem of planar graphs and planarization, and, in Section 4, we explain the proposed face routing in detail. In Section 5, a comparison of its performance with existing research through experiments is reported, and conclusions are drawn in Section 6.

## 2. Related Works

Early proposals for face routing methods using planar graphs include Greedy Face Greedy (GFG) [20], Compass Routing II [21], Greedy Perimeter Stateless Routing (GPSR) [18], Greedy Other Adaptive Face Routing (GOAFR+) [22], GOAFR++ [23], and Greedy Path Vector Face Routing (GPVFR) [24]. The methods forward a message along a sequence of faces intersecting with line $\bar{st}$ between the source (*s*) and the destination (*t*). A face change occurs when the edge of a face intersects with line $\bar{st}$. Messages are delivered based solely on local information and the faces are traversed sequentially. To improve performance, they are able to choose whether to pass through the intersecting edge or not when changing the face or to change how line $\bar{st}$ is set up and used. That is to say, they improve performance by changing the face after going through or without passing through the intersecting edge. They also enhance efficiency by using the initial setup of line $\bar{st}$ without changing during the entire routing process, or using a newly set up line $\bar{st}$ whenever a face is changed. Their performance also depends on the choice of traversal direction change, i.e., clockwise or counterclockwise. In short, early face routing improves performance and efficiency by determining how line $\bar{st}$ is set, when to change the face, which face to change to, and in which direction to explore the face is. Because they process routing using only local information, not global network information, routing information is not propagated beyond a single hop, thus maintaining locality. This locality means that the size of the routing table is determined according to the density of neighbors regardless of the network’s scale. Therefore, it can support scalability by not causing any degradation of performance, even when the network expands, and it can minimize information exchange between nodes because only the location information of neighbor nodes is used for routing. However, since they deliver the message using the planar graph connected with short edges, a problem with increasing hop count occurs when the message is delivered sequentially along the face boundary. In particular, in a high density WSN, the number of nodes to be passed through increases, resulting in sharp performance degradation.

ProgressFace [25] and Concurrent Face Routing (CFR) [26] have improved the performance and efficiency of early face routing by modifying how to forward the message instead of adjusting how to change the face. ProgressFace alleviates the problem of too many hops being taken if messages are forwarded in the wrong direction due to the unbalanced deployment of the nodes. It finds the direction with the smallest number of hops through an additional traversal step and improves routing efficiency by setting the forwarding direction to the way it finds, after which it sends the message. CFR focuses on the speed of message delivery and accelerates message propagation by sending messages concurrently in both directions of faces intersecting with line $\bar{st}$. It can send messages to the destination via the shortest possible route because it disseminates them through all available routes via the faces adjacent to line $\bar{st}$. Although it sends many duplicate messages to the network, it improves routing performance by sending messages at speeds similar to those sent by the shortest path. They have improved the performance, efficiency, and speed of routing by adjusting the search direction, using an additional traversal step, or forwarding duplicate messages. However, since they forward messages by sequentially exploring all the nodes constituting a face regardless of radio range, performance rapidly deteriorates due to the increased number of nodes that need to be traversed in high density WSNs.

In order to improve the forwarding efficiency by considering radio range, face routing using all of the node information within two hops has been proposed [19]. This improves routing efficiency by building a local full planar graph with the information of the 1- and 2-hop nodes, and directly forwarding the message to the most remote node within radio range using the generated graph. However, it is also unsuitable for high density WSNs due to the increased communication cost for transmitting the location information of such a large number of nodes within a 2-hop range and the performance degradation caused by the increased calculation cost due to the increased number of nodes.

## 3. The Problem with Planar Graphs and Planarization

**Figure 1:**
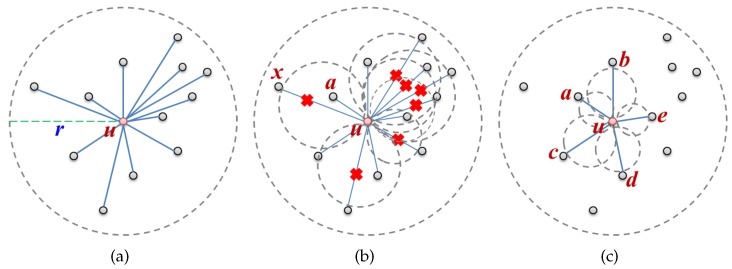
Planarization process: (**a**) local full network graph (*r*: radio range); (**b**) planarization; and (**c**) local Gabriel graph (GG).

GG and RNG are representative planar graphs constructed using only local information and do not divide a network. They are built by removing what might be intersecting edges among the edges between a message transfer node and its neighbors. Figure 1 shows the process of building a local GG: Figure 1a represents the local network graph of node *u* and Figure 1b depicts the planarization process that eliminates edges that do not fit the GG edge condition. An edge is removed if there is a node other than node *u* and a neighbor in the circle whose diameter is their edge. To illustrate this, in Figure 1b, node *a* exists in the circle within the edge diameter between nodes *u* and *x*, so edge(u,x) is removed. Figure 1c shows the planar graph generated after planarization. For the edge removal process according to the GG edge condition, long edges are removed and only short edges are left. Since the constructed planar graph consists of short edges, it causes a problem when forwarding a message sequentially along multiple nodes in the face in spite of being able to send it directly when in radio range.

**Figure 2:**
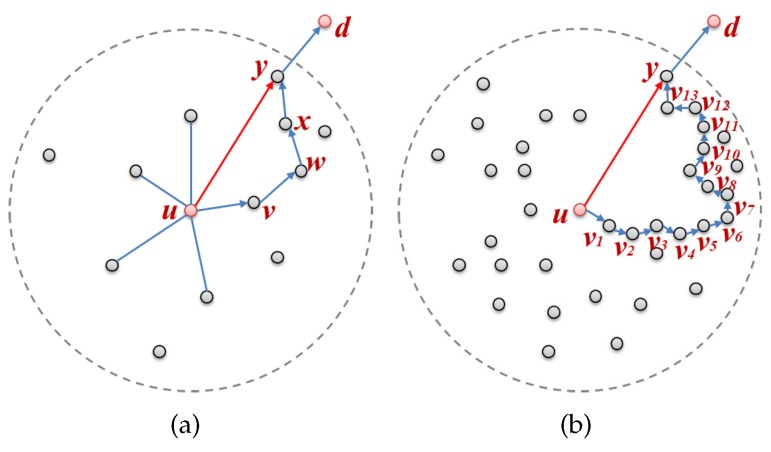
Face routing according to the node density: (**a**) a low density network; and (**b**) a high density network.

In Figure 2a, if nodes u,v,w,x,y, and *d* are on the routing path that make up the face, a problem with efficiency arises in that a message should have been transferred to node *y* after exploring nodes v,w, and *x*. Although node *y* is within radio range, the message cannot be forwarded from node *u* directly to node *y* because edge(u,y) was removed during planarization. Consequently, in a high density WSN, the inefficiency caused by this becomes even more exacerbated, as shown in Figure 2b; a message is transmitted through 15 hops ($u\to v_{1}\to \ldots \to v_{13}\to y\to d$) even though it could be transferred in only 2 hops (u→y→d). As the density of the nodes increases, the number of nodes within radio range increases, and, as this happens, the edges of the planar graph become shorter during planarization. The shortened edges intensify the inefficiency because they increase the number of nodes that need to be traversed when forwarding a message.

## 4. Transfer-Efficient Face Routing

In this section, we explain in detail the proposed face routing: transfer-efficient face routing (TEFR), which improves transfer efficiency by using the planar graph of the neighbor. Research on TEFR started with the notion that if a routing path can be known in advance within radio range, a message can be forwarded without travelling via intermediate nodes. Thus, if the routing path ABCDF can be determined in advance, it will be possible to efficiently send the message through a reduced number of hops by direct transmission from A to F. TEFR uses a local full planar graph that has been built with the planar graphs of neighbors to know the routing path within radio range in advance.

TEFR goes through three phases. In the first phase, location information is exchanged between nodes. In the second phase, the local planar graph is built using the received node information, and the generated graph information is transmitted to the message transfer node. Finally, the message transfer node generates the full planar graph within radio range with the collected planar graph information, finds the remote node using this, and then transmits the message.

TEFR is used in conjunction with greedy routing for message forwarding in void areas where greedy routing cannot deliver a message. We assumed that nodes are deployed randomly in a two-dimensional plane, that the link between nodes is reliable, and that their power status, their own position and the location of the source and destination of the message are known. We used a GG composed of a unit disk graph as a planar graph. We describe the exchange of location information between nodes and the construction of local planar graphs, and then we define the process of discovering the node to send a message to using the generated planar graphs of its neighbors. Finally, we analyze the performance of the proposed face routing.

### 4.1. The Exchange of Location Information

The first step of TEFR is to exchange location information between nodes. Since TEFR is a position-based routing method, it is necessary to collect the location information of the neighbors that is exchanged periodically using a beacon or aperiodically when there are topological changes. A beacon message consists of three fields:

- id: the local identifier of a node used to distinguish nodes within 2-hop range,
- $L_{x}$ and $L_{y}$: the *x* and *y* coordinates of a node indicating its location.

**Figure 3:**
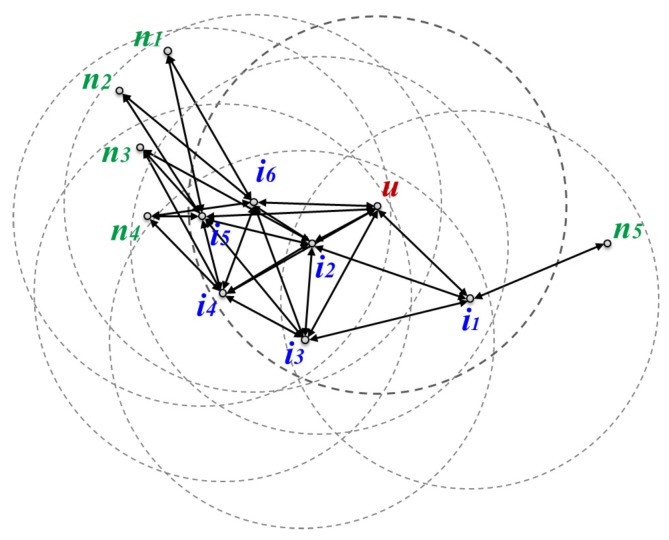
Beaconing: the exchange of position information between nodes.

Since the message transfer node in TEFR uses the planar graph of itself and its neighbors, it can receive position information on 2-hop nodes from its neighbors. Therefore, all node ids should be identified within 2-hop range. Figure 3 demonstrates the exchange of location information between nodes. Since message transfer node *u* can receive location information on $n_{1},n_{2},n_{3},n_{4}$, and $n_{5}$ located at 2 hops from neighbors, $i_{1},i_{2},i_{3},i_{4},i_{5}$, and $i_{6}$, node ids need to be distinguished within 2-hop range.

### 4.2. Build a Local Planar Graph

The second step of TEFR is to construct a local planar graph using the location information on the neighboring nodes and to transfer the information on the constructed planar graph to the message transfer node. The message transfer node and its neighbors generate their own local network graphs and their local GGs are built after planarization. Table 1 shows the edge list of the constructed local network graph of the message transfer node and its neighbors after exchanging location information between nodes in the network in Figure 3. Although the local network graph is part of the overall network graph, it is still possible to cause a loop across the entire network. Thus, even though the local network is tiny, it is necessary to remove any intersecting edges that might cause a loop by applying planarization.

**Figure 4:**
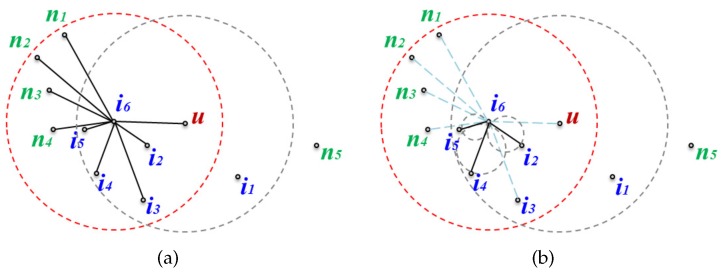
Local graphs of node $i_{6}$: (**a**) the local network graph; and (**b**) the local Gabriel graph (GG).

A local GG edge list is built for the message transfer node and its neighbors by removing edges that do not satisfy the GG edge condition from the edge list of the local network graphs to prevent looping. Figure 4 shows a local network graph and a local planar graph of node $i_{6}$ as an example of a planar graph built for the neighbors of a message transfer node. In Figure 4a, to perform planarization for node $i_{6}$, the GG edge condition is applied to all the edges connected to its neighbors. The resulting planar graph is shown in Figure 4b.

Planarization is performed independently on the message transfer node and all its neighbors. The pseudo-code running on each node for generating the GG edge list from the edge list of a local network graph is as follows.

**Algorithm 1** Generating the GG edge list from the edge list of a local network graph. LNG: edge list of the local network graph LGG: edge list of the local Gabriel graph **for** all edges in LNG
**do**    **if** (there exists no node in the circle with edge’s diameter) **then**    add edge to LGG    **end if** **end for**

The algorithm generates the GG edge list by selecting those edges meeting the GG edge condition among all edges on the local network graph. Table 2 shows the GG edge lists constructed by applying Algorithm 1 to the local network graph edge lists in Table 1. The strikethroughs in Table 2 represent edges that were deleted during planarization, so are not GG edges.

Since TEFR distributes planarization to the message transfer nodes’ neighbors, it is possible to solve the problem that the load is concentrated on the message transfer nodes, which makes face routing using 2-hop node information so inefficient. Moreover, because the message transfer node only receives information of the planar graph nodes rather than of all neighboring nodes, the amount of position information to be transmitted can be reduced. In addition, the changes are transmitted to the message transfer node each time a node is added or removed only if the planar graph is affected due to a change in topology. Therefore, TEFR improves efficiency and minimizes the amount of location information that needs to be transferred.

**Figure 5:**
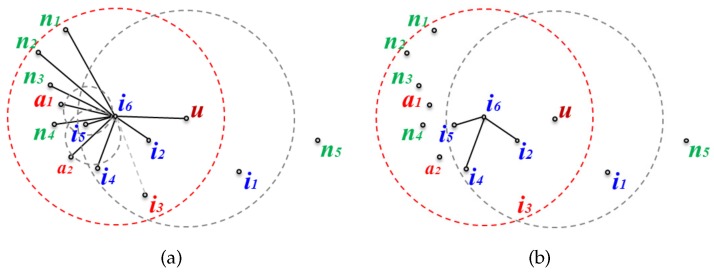
Topological changes that do not affect the planar graph: (**a**) changed local network graph, and (**b**) (a)’s planar graph.

**Figure 6:**
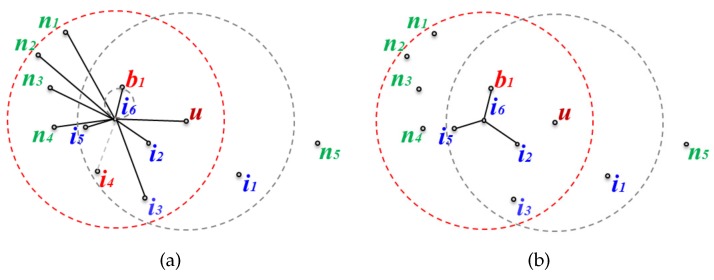
Topological changes that affect the planar graph: (**a**) changed local network graph, and (**b**) (a)’s planar graph.

Figure 5 and Figure 6 show two cases where the changed node information is either sent or not sent to the message transfer node when a node is added or removed. Figure 5 shows the case where the topology is changed but the changes do not affect the planar graph, while Figure 6 shows the opposite case to Figure 5 where the changed node information should be sent to the message transfer node.

Figure 5a is the local network where new nodes $a_{1}$ and $a_{2}$ are added, and node $i_{3}$ is deleted in the network of Figure 4a. Even if nodes $a_{1}$ and $a_{2}$ are added but the edges between node $i_{6}$ and them are deleted during planarization, this does not affect the existing planar graph. Furthermore, even though node $i_{3}$ is removed from the network since $edge(i_{6},i_{3})$ has already been deleted from the existing planar graph due to not fitting the GG edge condition, this also does not affect the existing planar graph. Figure 5b shows the planar graph after topological changes have occurred. Since it is the same as before the network change, the changed node information is not transmitted even though there have been topological changes.

Figure 6 shows the case where a topological change affects the planar graph. Figure 6a depicts the local network where new node $b_{1}$ is added to the network of Figure 4a, and $i_{4}$ is deleted. $edge(i_{6},b_{1})$ becomes a new edge on the planar graph according to the GG edge condition, and $edge(i_{6},i_{4})$, which was an edge on the existing planar graph, is removed because the node has been deleted (Figure 6b shows the planar graph after adding and removing the nodes). Since the existing planar graph has been changed, the changed planar graph information on node $i_{6}$ must be sent to the message transfer node.

### 4.3. Remote Node Selection and Message Forwarding

The last step in the TEFR method is to discover the node located at the most distant hop on a routing path within radio range using the information on the planar graphs of the neighbors at the message transfer node, and then send the message. The message transfer node generates an edge list of the full planar graph within radio range using the local planar graph information received from the neighbor to determine which node the message is to be transferred to.

**Figure 7:**
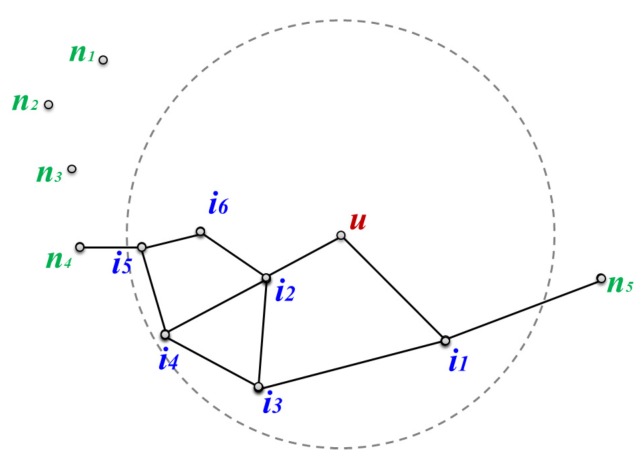
Local full Gabriel graph (GG) within radio range of node *u*.

Table 3 shows the full GG edge list within radio range built with the planar graph information of its neighbors at message transfer node *u* in Figure 3. Each item in the edge list consists of the start node id, the end node id, and their x,y coordinates: p_id, the immediate neighbor of the message transfer node, is the node id whose local planar graph is sent to the message transfer node and is the starting node of the edge; n_id represents the opposite side node of the edge; and $L_{x}$ and $L_{y}$ represent the location information for the p_id and n_id nodes. When the start and end nodes of all edges in the list are connected, a full planar graph within radio range of a message transfer node is generated. Figure 7 shows the local full GG within radio range of message transfer node *u*, which is constructed using the edges in Table 3.

**Figure 8:**
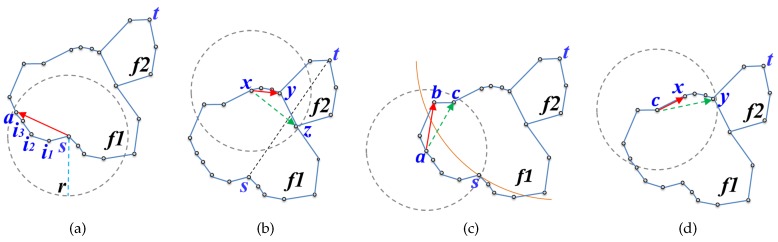
Four cases of node selection: (**a**) routing within the same face, (**b**) a face change occurs, (**c**) return to greedy mode, and (**d**) sequential forwarding. The arc in (**c**) signifies the distance between the destination (*t*) and node *s* where the face routing started.

TEFR uses the generated edge list to determine the node to send the message to. In the discovery process, four cases need to be considered, as shown in Figure 8. The first three cases are for direct forwarding to the remote node within radio range, and the last case is for sequential forwarding applied when the remaining power of the message transfer node is insufficient. For direct transmission, the first is when message forwarding occurs within the same face, the second is when a face change occurs, and the third is when the routing mode is switched from face routing to greedy routing.

In the first case, a message is sequentially transferred along the face within radio range and the change of face or routing mode does not occur during the routing. In this instance, the node located at the last hop of the face boundary within radio range is selected as the node where the message is to be sent to. Figure 8a shows the case where a message is forwarded within the same face. TEFR selects node *a* located at the farthest hop within radio range of message transfer node *s* as the most remote node, and the message is sent to it.

The second case arises when a face change occurs while the message is forwarded within radio range because the line from the source to the destination and a face boundary edge intersect. In this case, TEFR transmits the message to the node where the intersecting line occurs, not the most distant hop node within radio range. After the selected node receives the message, it changes the face and routing is continued. Figure 8b shows the case where the face is changed because the intersection between line $\bar{st}$ and edge(y,z) occurs while traveling to the face within radio range of message transfer node *x*. If the message is sent to the most distant node *z* without considering the face change at message transfer node *x*, a loop occurs because the face change does not execute. Therefore, message transfer node *x* forwards the message to node *y* where the face change occurs, after which node *y* changes the face and then continues routing.

The third case happens when it is necessary to switch to greedy routing while traveling to a face within radio range. TEFR immediately switches from face routing to greedy routing when greedy routing is enabled and it forwards a message according to greedy routing. Figure 8c shows the case where the routing mode needs to be switched from face routing to greedy routing during face traversal within radio range. Message transfer node *a* forwards the message to the first node *b* closer to the destination (*t*) than node *s* where face routing started without sending a message to the farthest hop node *c* on the face boundary within radio range. After receiving the message, node *b* switches to greedy routing and performs message forwarding.

The last case occurs when the message transfer node has low residual power. A message is sent sequentially along the face boundary instead of being forwarded to the remote node. Because energy consumption increases as the distance to be transmitted increases in wireless communication [27], the message transfer node sends the message to its nearest neighbor when the energy level is low to minimize power consumption. This extends the lifetime of the node and consequently prolongs the lifetime of the WSN. By selectively transmitting a message to the remote node or nearby nodes according to the remaining power, transfer efficiency can be improved while using the energy of each node in a balanced manner. Figure 8d shows the case where the message transfer node has less energy than the threshold for direct transmission. Message transfer node *c* can forward a message directly to remote node *y*, but it sends the message to nearest node *x* to minimize energy consumption.

Since TEFR can determine the routing path in advance within radio range using the full GG edge list, it can establish the most remote node where a message can be transferred most efficiently, and a message can be sent directly to the selected node. That is to say, TEFR can directly send a message without journeying via intermediate nodes by simulating routing internally using the edge list as in Table 3 and selecting a remote node in the same way as actually traveling the nodes constituting the face within radio range.

**Figure 9:**
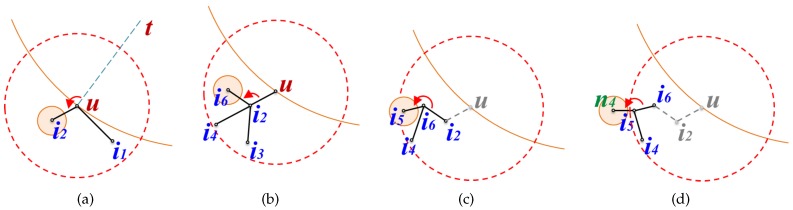
Phases of TEFR node selection: (**a**) the start of simulating face routing, (**b**,**c**) select the next nodes of nodes $i_{2}$ and $i_{6}$, respectively, and (**d**) the termination of node selection. The arc signifies the distance between the destination (*t*) and node *u* where face routing started.

Figure 9 shows the internal process of node selection at the message transfer node required to send a message, which corresponds to the case of forwarding a message within the same face as in Figure 8a. All the processes in Figure 9 are performed internally at message transfer node *u*, and after the process is finished, a message is forwarded to the selected node.

Figure 9a shows the beginning of the face routing simulation to establish the node where the message is to be sent to. Face routing is started at message transfer node *u* because there is no node closer than itself to the destination (*t*) among the neighbors. Node *u* selects $i_{2}$ as the next node to be explored by applying the left-hand rule based on line $\bar{ut}$ among neighboring nodes $i_{1}$ and $i_{2}$ in the GG edge list in Table 3.

In Figure 9b, node $i_{2}$ selects $i_{6}$ as the next node to explore according to the left-hand rule, and checks whether it is within radio range of node *u*, whether the routing mode should be switched to greedy routing, and whether an intersecting edge occurs. Node $i_{6}$ is located within radio range of node *u*, is farther from the destination than node *u* where face routing started, and $edge(i_{2},i_{6})$ does not intersect line $\bar{ut}$. Thus, $i_{6}$ is selected as the next node to navigate to. In Figure 9c, $i_{5}$ is selected as the next node to travel to from node $i_{6}$ in the face routing procedure according to the same condition as node $i_{6}$.

In Figure 9d, node $n_{4}$, which is the next node along from node $i_{5}$, is beyond the radio range of message transfer node *u*. Therefore, message transfer node *u* terminates the node discovery process and selects $i_{5}$ as the target node to send the message to. Message transfer node *u* improves the transfer efficiency by sending a message directly to the most remote node $i_{5}$ without actually passing through the intermediate nodes $i_{2}$ and $i_{6}$. The pseudo-code for selecting the most remote node using the GG full edge list of a message transfer node and sending a message is as Algorithm 2.

### 4.4. Analysis

TEFR utilizing the local planar graphs of the message transfer node’s neighbors needs more location information compared with legacy face routing using local information but uses less position information than face routing using the information on all nodes within 2-hop range. Equation (1) compares the quantity of location information transmitted to a message transfer node. In the equation, the left-hand side is legacy face routing, the middle is TEFR, and the right-hand side is face routing using 2-hop node information:

(1) $$n\times m\leq n\times m+n\times \left(\left(1-\lambda \right)\times n\right)\times m\leq n\times m+n^{2}\times m,$$

where *n* is the average number of neighboring nodes per node, *m* is the data size of location information for one node, and λ is the ratio of nodes removed in planarization (0≤λ<1).

**Algorithm 2** Selecting the most remote node using the GG edge list and sending a message.lhn : a node according to left hand rulectn : a current traversal nodemtn : a message transfer nodefrn : a node where face routing startedthd : the power threshold for direct transmission
select lhn of ctn from GG edge list**if** ( the power level of ctn < thd ) **then**    send message to lhn, exit**end if****while** (lhn is located within radio range of mtn) **do**    **if** (lhn is closer to the destination than frn) **then**        change mode to greedy routing        send message to lhn, exit    **else if** (the edge between lhn and ctn intersects $\bar{st}$) **then**        send message to ctn, exit    **else**        set ctn with lhn        select lhn of ctn from GG edge list    **end if****end while**send message to ctn, exit

In Equation (1), in legacy face routing, a message transfer node only receives location information on the average number of neighboring nodes, while TEFR receives the location information on the immediate neighbors and their planar graph nodes after planarization. Face routing using 2-hop node information receives the location information on the immediate neighbors and all of their neighbors as well. The amount of location information required for TEFR is the same as that for face routing using 2-hop node information when nodes are not removed in the planarization step (λ = 0) and becomes close to that of legacy face routing as the number of removed nodes increases.

In a high density WSN, TEFR does not rapidly increase the amount of position information to be transmitted because, even if the number of nodes increases, those removed by planarization increases and λ becomes bigger as the density of the nodes increases. TEFR uses the location information on more nodes than the legacy face routing to improve transfer efficiency, but it reduces the amount of position information in comparison with face routing using 2-hop node information because message transfer nodes only receive the information on the planar graph nodes of their neighbors.

The space complexity of a node for each face routing can be determined using Equation (1). O(n), $O(n^{2})$, and $O(n^{2})$ are respectively the space complexity of each node for legacy face routing using local information, face routing using 2-hop node information, and TEFR, where *n* is the number of deployed nodes.

Equation (2) compares the end-to-end delay of face routing within radio range. The first formula is the delay of legacy face routing, the second is that of face routing using 2-hop node information, and the last is that of TEFR:

(2) $$\begin{array}{ccc} \left(n-1\right) & \times & (D_{ew}+D_{t}+k\times D_{pp}+D_{ps}+D_{q})\\ & & D_{ew}+D_{t}+k^{2}\times D_{pp}+D_{ps}+D_{q}\\ & \geq & D_{ew}+D_{t}+k\times D_{pp}+D_{ps}+D_{q}. \end{array}$$

End-to-end delay can be divided into propagation delay ($D_{ew}$), transmission delay ($D_{t}$), processing delay ($D_{p}$), and queueing delay ($D_{q}$) [28]; we divide $D_{p}$ into the time ($D_{pp}$) required to execute planarization for individual neighboring nodes and the time ($D_{ps}$) for determining the node to which the message is to be sent. Furthermore, in the formulae, *n* is the number of nodes on the face boundary to be traveled within radio range (n>1) and *k* represents the average number of neighboring nodes.

In Equation (2), since legacy face routing needs to sequentially travel the nodes on the face boundary, the end-to-end delay is highly influenced by the number of nodes constituting the face. However, TEFR and face routing using 2-hop node information are not affected by the number of nodes constituting the face because the message is forwarded directly to the most remote node within radio range.

Since face routing using 2-hop node information performs planarization on all edges within 2-hop range of the message transfer node, the execution time of planarization is required for the square of the number of neighboring nodes. On the other hand, TEFR only requires the execution time for planarization on the local nodes because it performs planarization independently on each neighboring node. As the number of deployed nodes increases, the performance of face routing using 2-hop node information decreases rapidly due to the increase in planarization time caused by squaring the number of neighboring nodes. In contrast, TEFR can forward a message without an abrupt decrease in performance because the planarization time is gradually prolonged in proportion to the number of neighboring nodes.

The time complexity of the computation time for message transmission can be inferred from Equation (2). Each node used for legacy face routing using local information, face routing using 2-hop node information, and TEFR can determine the next node for forwarding a message in O(k), $O(k^{2})$, and O(k) computation time respectively, where *k* is the number of deployed nodes.

## 5. Performance Evaluation

In this section, we compare the performance of legacy face routing, face routing using 2-hop node information, and the face routing proposed in this paper. In order to compare the independent performance of each, we implemented a simulation with only face routing except greedy routing.

### 5.1. Simulation Environment

**Figure 10:**
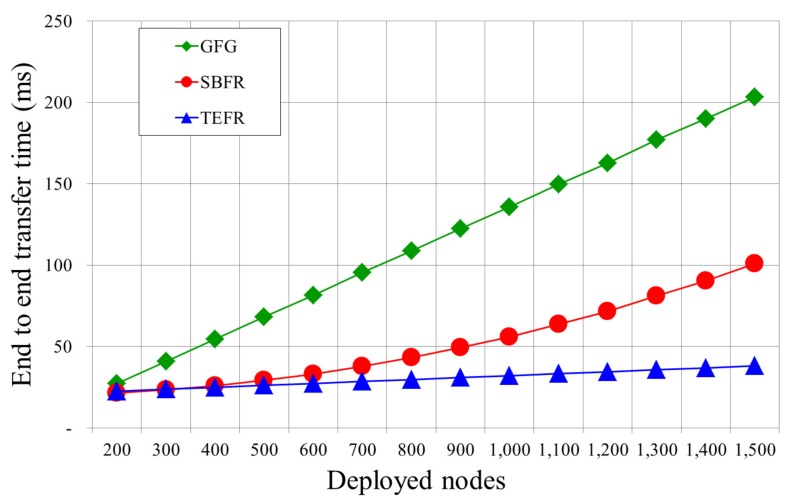
Transfer time according to the number of deployed nodes (radio range: 100 m).

**Figure 11:**
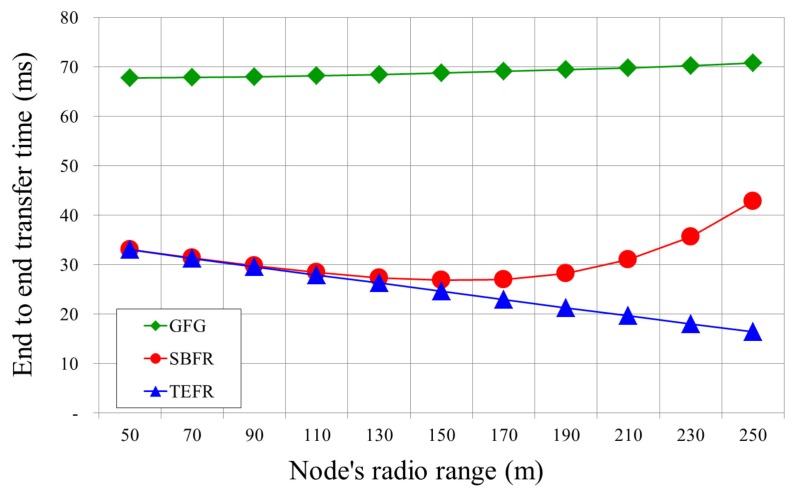
Transfer time according to radio range (deployed nodes: 500).

We simulated the performance of the proposed face routing using the network simulator Qualnet 4.0 (Scalable Network Technologies, US State) [29]. The radio range of the sensor node was 50∼250 m, and 200∼1500 nodes were randomly deployed in a space of 1000 × 1000 m${}^{2}$. Simulations were performed by increasing the radio range by 20 m intervals and increasing the number of deployed nodes by 100 units. In other words, for the simulation in Figure 11, we experimented with each case by increasing the radio range from 50 m to 250 m in 20 m increments in an environment with 500 deployed nodes (radio range: 50 m, 70 m, 90 m, …, 250 m). For the simulation shown in Figure 10, when the radio range was 100 m, experiments were carried out by increasing the number of nodes by 100 units from 200 to 1500 (deployed nodes: 200, 300, 400, …, 1500). The network model was a unit disk graph connected from the source to the destination, and GG was used as the planar graph. The network unit disk graph is an intersection graph, in which all nodes have the same radio range, and two nodes are connected if and only if they are within the unit-distance of each other. The simulation time was set to 100 s and each sensor node transmitted its position to its neighbors. All the results were the average of each simulation experiment repeated 100 times. In the simulation, Greedy Face Greedy Routing (GFG) [20] as legacy face routing using local information, Shortcut Based Face Routing (SBFR) [19] using all node information within 2-hop range, and TEFR using the planar graphs of neighbors were used. The overall simulation environments are given in Table 4.

### 5.2. Simulation Results

Figure 10 shows a graph comparing the time at which the message was forwarded from the source to the destination according to the number of deployed nodes. With GFG, as the number of deployed nodes increases, the number of nodes to be traveled increases because the number of nodes constituting a face increases. Thereby, the transfer time increases gradually in proportion to the increased number of nodes. With SBFR, since a message is sent directly to the most remote node within radio range, even if the number of nodes increases, the time for forwarding a message is constant. However, since it performs planarization on all nodes within 2-hop range of a message transfer node, as the number of nodes increased, the computation time increased, resulting in the message transfer time, which includes the computation time, becoming longer. Since TEFR sends a message directly to the most remote node within radio range and the generation of the planar graph is performed in a distributed manner at the neighbor node, the increase in the number of nodes has little effect on the transfer time. From the experimental results, we can confirmed that the transfer time of TEFR was hardly affected by an increase in the number of nodes.

Figure 11 shows a graph comparing the transfer time from the source to the destination according to changes in radio range. With GFG, even if the radio range is widened, it is necessary to sequentially travel along the nodes connected with short edges regardless of the radio range. Thus, the transfer time did not decrease even when the radio range was expanded. With SBFR, as the radio range increases, a message can be directly sent to a node located at a longer distance, thereby reducing the transfer time. However, as the radio range is widened, the number of nodes within radio range increases and the construction time of the planar graph increases accordingly. As the radio range exceeds a certain distance, the wider the radio range, the longer the transfer time. With TEFR, even if the number of nodes to be planarized increases due to an increment in radio range, the transfer time is not significantly affected because planarization is distributed to the neighbors of the message transfer node, and the message can also be sent directly to the node farthest away. As a result, as the radio range increased, the transfer time decreased.

**Figure 12:**
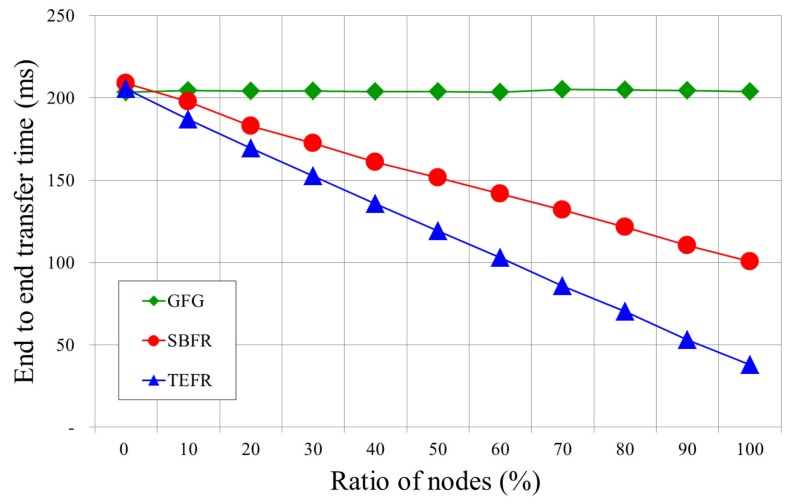
Transfer time according to ratio of nodes capable of direct transmission (radio range: 100 m, deployed nodes: 1500).

Figure 12 shows the transfer time of each face routing according to the ratio of the node that can forward a message directly to the remote node in an experiment to consider the power state of the node. In practice, SBFR does not take into account the power state of the node, but in order to compare the transfer time in this simulation, it sends a message sequentially when the message transfer node has less power than the threshold. Since GFG transfers a message sequentially along the face boundary, the transfer times are similar regardless of the proportion of nodes performing direct transmission. However, in SBFR and TEFR, the number of nodes forwarding a message to the remote node also increases as the ratio of nodes capable of direct transmission increases, resulting in a decrease in end-to-end transfer time.

The ratio of nodes is 0% when all of the deployed nodes send messages sequentially along the face boundary. When the ratio of nodes is 100%, all nodes are able to forward messages directly to the remote node. TEFR shows performance similar to GFG or SBFR when the nodes send messages sequentially. In addition, as the number of nodes capable of direct transmission increases, it performs better than GFG and SBFR due to the increased ratio of forwarding a message directly to the remote node and the faster computation time for routing than SBFR.

**Figure 13:**
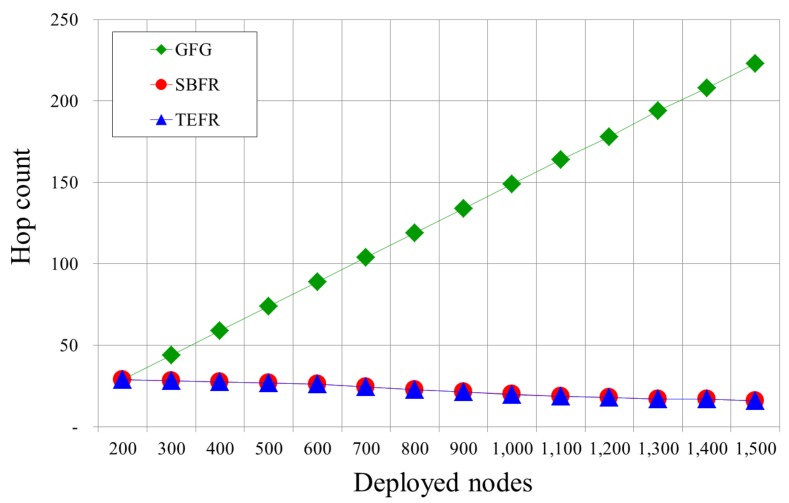
Hop counts according to the number of deployed nodes (radio range: 100 m).

Figure 13 is a graph of the total number of hops from the source to the destination by the number of deployed nodes. With GFG, as the number of deployed nodes increases, the edges on the planar graph become shorter. Therefore, the number of hops to journey from the source to the destination increases. Since SBFR and TEFR send a message directly to the most remote node within radio range, the number of hops rarely changes even if the number of nodes increases in the same radio range. SBFR and TEFR show similar hop counts because the number of hops is not related to computation time when constructing a planar graph.

**Figure 14:**
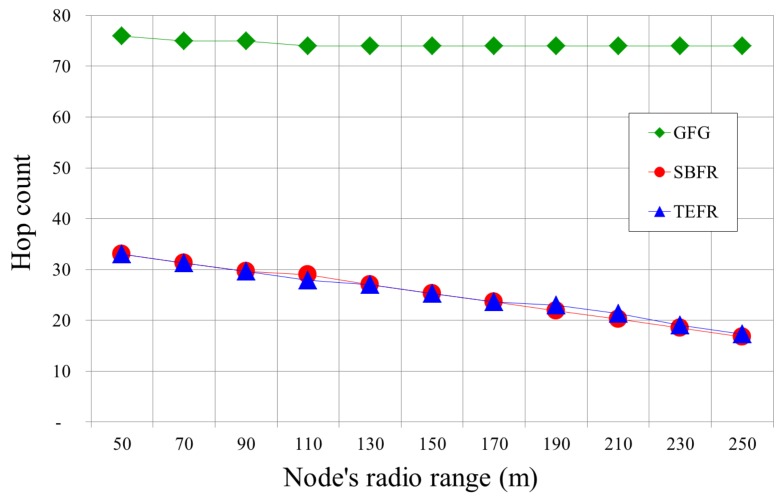
Hop counts according to radio range (deployed nodes: 500).

Figure 14 compares the number of hops with a change in radio range. With GFG, even if the node that the message can be directly forwarded to is further away due to the increased radio range, since long edges are removed in planarization and a message is forwarded along short edges, the radio range expansion does not affect the number of hops. In SBFR and TEFR, as the radio range increases, the number of hops decreases because they can transfer a message directly to a node that is further away, which was confirmed by the simulation results.

**Figure 15:**
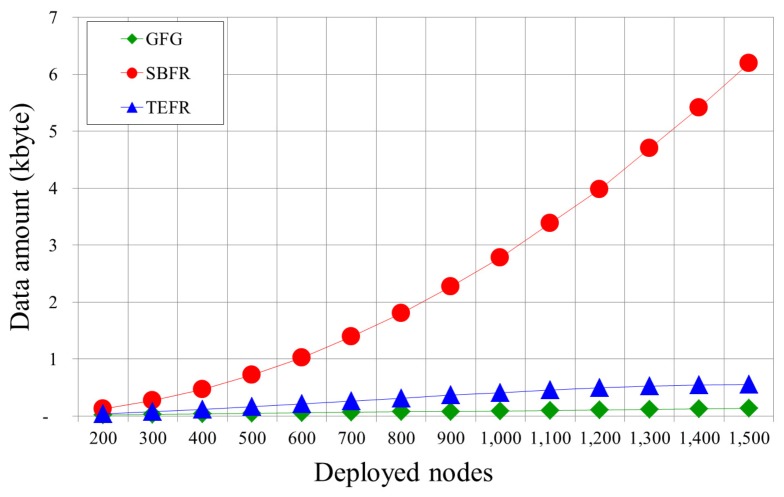
The quantity of transferred location information according to the number of nodes (radio range: 100 m).

Figure 15 depicts a comparison of the amount of location information to be transmitted as the number of nodes increases within the same radio range. Because GFG receives and uses only the position information of the message transfer node’s neighbors, as the number of deployed nodes increases, the number of neighboring nodes increases, and so the amount of the location information to be transmitted gradually increases. With SBFR, since the location information on all nodes within 2-hop range must be transmitted to the message transfer node, the amount of location information to be transmitted sharply increases as the number of nodes increases. In other words, when the average number of neighboring nodes in radio range is *n*, the message transfer node receives the position information on $n+n^{2}$ nodes since it receives the location information on *n* immediate neighboring nodes and *n* neighboring nodes for each of them. With TEFR, since the position information on its neighbors and their planar graph nodes is transmitted to the message transfer node, the amount of position information increases further as the number of nodes increases compared to GFG. However, unlike SBFR, the amount of location information does not increase rapidly because only the information of the nodes after planarization is transmitted. As the number of deployed nodes increases, the number of removed nodes in planarization also increases. Therefore, the amount of location information does not increase sharply even if the number of nodes to be deployed increases.

**Figure 16:**
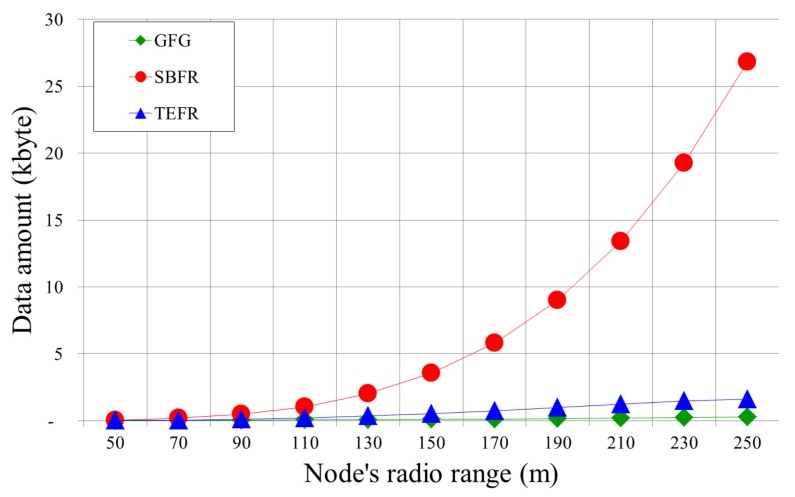
The quantity of transferred location information according to the radio range (Deployed nodes: 500).

Figure 16 shows changes in the amount of location information as radio range increases. With GFG, as the radio range increases, the number of the neighbor increases and the amount of location information to be transferred increases accordingly. With SBFR, as the radio range increases, more neighboring nodes transmit position information because of the much larger number of nodes in 2-hop range. Thus, as the radio range is expanded, the amount of the location information to be transmitted increases rapidly. With TEFR, even if the radio range increases, since only the location information on some neighbor nodes of the planar graph after the planarization is transmitted to the message transfer node, the amount of the location information is not increased by as much as with SBFR.

The experimental results show that the proposed face routing did not increase the transfer time, unlike with GFG and SBFR, even when the number of nodes increased, and the transfer time lessened as the radio range increased. A message was able to be sent to the destination within a certain number of hops regardless of the number of deployed nodes as long as the network was the same size. As the radio range became wider, the hop count was gradually reduced because a message can be directly forwarded to a node located at a longer distance away. In comparison with face routing using local information, more position information needs to be transmitted because the planar graph information of the neighbor is additionally used. However, since the neighbors of the message transfer node only transmit information on some nodes after planarization, it uses less location information than face routing using 2-hop node information.

Through our experimental results, we can see that the proposed face routing was able to forward a message at a faster and more constant rate in a high density WSN than the existing face routing methods, and it was able to forward messages to the destination within a certain number of hops. Furthermore, as the radio range was enlarged, the transfer time was reduced because the messages were forwarded by traveling via a smaller number of nodes from the source to the destination. Therefore, transfer efficiency can be further improved by adjusting the radio range when applying the proposed face routing in practice.

## 6. Conclusions

In this paper, we propose a new face routing to improve transfer efficiency by forwarding a message directly to the most distant hop node on the routing path within radio range using the planar graphs of neighbors. Since traditional face routing transfers a message using a planar graph to prevent a loop, a problem with decreased efficiency occurs as a result of forwarding a message sequentially along the face boundary composed of short edges although it can be delivered directly. In order to solve this problem, face routing that forwards a message directly to the most remote node within radio range using all of the node information within 2-hop range has been proposed, but this makes message transfer nodes receive too much location information and requires a great deal of computation for network graph generation and planarization. In a high density network, it causes another problem in that the processing time increases rapidly and a huge load is concentrated on the message transfer nodes.

The proposed face routing distributes loads and reduces message transfer time by using the planar graph information of the message transfer node’s neighbors. It distributes the construction and planarization process of the network graph to the neighbors and reduces the amount of position information to be transmitted through sending only the information concerning the planar graph nodes. Furthermore, it minimizes traffic and computation time for routing by transmitting the location information of changed nodes irrespective of topological changes and only when the planar graph is changed. These characteristics make it suitable for use in high density WSNs. Through performance evaluation, we confirmed that the proposed scheme improved transfer efficiency compared to the existing face routing methods. In future research, we would like to study a method involving end-to-end message forwarding within a limited timeframe regardless of the density of the deployed nodes and how to efficiently forward a message in the real environment where the link is unreliable.

## Figures and Tables

**Table 1:** The local network graph edge lists of message transfer node *u* and its neighbors in Figure 3.

Node *u*	Node $i_{1}$	Node $i_{2}$	Node $i_{3}$	Node $i_{4}$	Node $i_{5}$	Node $i_{6}$
*u*, $i_{1}$	$i_{1}$, *u*	$i_{2}$, *u*	$i_{3}$, *u*	$i_{4}$, *u*	$i_{5}$, *u*	$i_{6}$, *u*
*u*, $i_{2}$	$i_{1}$, $i_{2}$	$i_{2}$, $i_{1}$	$i_{3}$, $i_{1}$	$i_{4}$, $i_{2}$	$i_{5}$, $i_{2}$	$i_{6}$, $i_{2}$
*u*, $i_{3}$	$i_{1}$, $i_{3}$	$i_{2}$, $i_{3}$	$i_{3}$, $i_{2}$	$i_{4}$, $i_{3}$	$i_{5}$, $i_{3}$	$i_{6}$, $i_{3}$
*u*, $i_{4}$	$i_{1}$, $n_{5}$	$i_{2}$, $i_{4}$	$i_{3}$, $i_{4}$	$i_{4}$, $i_{5}$	$i_{5}$, $i_{4}$	$i_{6}$, $i_{4}$
*u*, $i_{5}$		$i_{2}$, $i_{5}$	$i_{3}$, $i_{5}$	$i_{4}$, $i_{6}$	$i_{5}$, $i_{6}$	$i_{6}$, $i_{5}$
*u*, $i_{6}$		$i_{2}$, $i_{6}$	$i_{3}$, $i_{6}$	$i_{4}$, $n_{3}$	$i_{5}$, $n_{1}$	$i_{6}$, $n_{1}$
		$i_{2}$, $n_{4}$		$i_{4}$, $n_{4}$	$i_{5}$, $n_{2}$	$i_{6}$, $n_{2}$
					$i_{5}$, $n_{3}$	$i_{6}$, $n_{3}$
					$i_{5}$, $n_{4}$	$i_{6}$, $n_{4}$

**Table 2:** The local planar graph edge lists of node *u* and its neighbors in Figure 3.

Node *u*	Node $i_{1}$	Node $i_{2}$	Node $i_{3}$	Node $i_{4}$	Node $i_{5}$	Node $i_{6}$
*u*, $i_{1}$	$i_{1}$, *u*	$i_{2}$, *u*	i3, u	i4, u	i5, u	i6, u
*u*, $i_{2}$	i3, i2	i2, i1	$i_{3}$, $i_{1}$	$i_{4}$, $i_{2}$	i5, i2	$i_{6}$, $i_{2}$
u, i3	$i_{1}$, $i_{3}$	$i_{2}$, $i_{3}$	$i_{3}$, $i_{2}$	$i_{4}$, $i_{3}$	i5, i3	i6, i3
u, i4	$i_{1}$, $n_{5}$	$i_{2}$, $i_{4}$	$i_{3}$, $i_{4}$	$i_{4}$, $i_{5}$	$i_{5}$, $i_{4}$	i6, i4
u, i5		i2, i5	i3, i5	i4, i6	$i_{5}$, $i_{6}$	$i_{6}$, $i_{5}$
u, i6		$i_{2}$, $i_{6}$	i3, i6	i4, n3	i5, n1	i6, n1
		i2, n4		i4, n4	i5, n2	i6, n2
					i5, n3	i6, n3
					$i_{5}$, $n_{4}$	i6, n4

**Table 3:** The GG full edge list of node *u* within radio range in Figure 3.

p_id	n_id	p_id		n_id		p_id	n_id	p_id		n_id	
		$L_{x}$	$L_{y}$	$L_{x}$	$L_{y}$			$L_{x}$	$L_{y}$	$L_{x}$	$L_{y}$
*u*	$i_{2}$	$x_{u}$	$y_{u}$	$x_{i_{2}}$	$y_{i_{2}}$	$i_{3}$	$i_{2}$	$x_{i_{3}}$	$y_{i_{3}}$	$x_{i_{2}}$	$y_{i_{2}}$
*u*	$i_{1}$	$x_{u}$	$y_{u}$	$x_{i_{1}}$	$y_{i_{1}}$	$i_{3}$	$i_{4}$	$x_{i_{3}}$	$y_{i_{3}}$	$x_{i_{4}}$	$y_{i_{4}}$
$i_{1}$	*u*	$x_{i_{1}}$	$y_{i_{1}}$	$x_{u}$	$y_{u}$	$i_{4}$	$i_{3}$	$x_{i_{4}}$	$y_{i_{4}}$	$x_{i_{3}}$	$y_{i_{3}}$
$i_{1}$	$i_{3}$	$x_{i_{1}}$	$y_{i_{1}}$	$x_{i_{3}}$	$y_{i_{3}}$	$i_{4}$	$i_{2}$	$x_{i_{4}}$	$y_{i_{4}}$	$x_{i_{2}}$	$y_{i_{2}}$
$i_{1}$	$n_{5}$	$x_{i_{1}}$	$y_{i_{1}}$	$x_{n_{5}}$	$y_{n_{5}}$	$i_{4}$	$i_{5}$	$x_{i_{4}}$	$y_{i_{4}}$	$x_{i_{5}}$	$y_{i_{5}}$
$i_{2}$	*u*	$x_{i_{2}}$	$y_{i_{2}}$	$x_{u}$	$y_{u}$	$i_{5}$	$i_{6}$	$x_{i_{5}}$	$y_{i_{5}}$	$x_{i_{6}}$	$y_{i_{6}}$
$i_{2}$	$i_{6}$	$x_{i_{2}}$	$y_{i_{2}}$	$x_{i_{6}}$	$y_{i_{6}}$	$i_{5}$	$n_{4}$	$x_{i_{5}}$	$y_{i_{5}}$	$x_{n_{4}}$	$y_{n_{4}}$
$i_{2}$	$i_{4}$	$x_{i_{2}}$	$y_{i_{2}}$	$x_{i_{4}}$	$y_{i_{4}}$	$i_{5}$	$i_{4}$	$x_{i_{5}}$	$y_{i_{5}}$	$x_{i_{4}}$	$y_{i_{4}}$
$i_{2}$	$i_{3}$	$x_{i_{2}}$	$y_{i_{2}}$	$x_{i_{3}}$	$y_{i_{3}}$	$i_{6}$	$i_{5}$	$x_{i_{6}}$	$y_{i_{6}}$	$x_{i_{5}}$	$y_{i_{5}}$
$i_{3}$	$i_{1}$	$x_{i_{3}}$	$y_{i_{3}}$	$x_{i_{1}}$	$y_{i_{1}}$	$i_{6}$	$i_{2}$	$x_{i_{6}}$	$y_{i_{6}}$	$x_{i_{2}}$	$y_{i_{2}}$

**Table 4:** Simluation environment.

Parameters	Values
Simulator	Qualnet 4.0
The radio range of the sensor node	50∼250 m (increased by 20 m)
The number of sensor nodes	200∼1500 nodes (increased by 100 nodes)
Node placement	Random
Network size	1000 × 1000 m${}^{2}$
Network model	unit disk graph
Routing protocols	GFG, SBFR, TEFR
Planar graph	Gabriel graph
Simulation time	100 s
Number of Simulation Executions	100 times

## References

[1] Othman M.F., Shazali K. (2012). Wireless sensor network applications: A study in environment monitoring system. Procedia Eng..

[2] Rawat P., Singh K.D., Chaouchi H., Bonnin J.M. (2014). Wireless sensor networks: A survey on recent developments and potential synergies. J. Supercomput..

[3] Bapat V., Kale P., Shinde V., Deshpande N., Shaligram A. (2017). Wsn application for crop protection to divert animal intrusions in the agricultural land. Comput. Electron. Agric..

[4] Suzuki M., Saruwatari S., Kurata N., Morikawa H. A high-density earthquake monitoring system using wireless sensor networks. Proceedings of the 5th International Conference on Embedded Networked Sensor Systems.

[5] Pantazis N.A., Nikolidakis S.A., Vergados D.D. (2013). Energy-efficient routing protocols in wireless sensor networks: A survey. IEEE Commun. Surv. Tutor..

[6] Ahmadi A., Shojafar M., Hajeforosh S.F., Dehghan M., Singhal M. (2014). An efficient routing algorithm to preserve k-coverage in wireless sensor networks. J. Supercomput..

[7] Santos R., Edwards A., Verduzco A. A geographic routing algorithm for wireless sensor networks. Proceedings of the Electronics, Robotics and Automotive Mechanics Conference.

[8] Ruhrup S. (2009). Theory and practice of geographic routing. Ad Hoc and Sensor Wireless Networks: Architectures, Algorithms and Protocols.

[9] Al-Karaki J.N., Kamal A.E. (2004). Routing techniques in wireless sensor networks: A survey. IEEE Wirel. Commun..

[10] Seada K., Helmy A. (2004). Geographic Protocols in Sensor Networks.

[11] Stojmenovic I. (2002). Position-based routing in ad hoc networks. IEEE Commun. Mag..

[12] Zonouz A.E., Xing L., Vokkarane V.M., Sun Y.L. (2014). Reliability-oriented single-path routing protocols in wireless sensor networks. IEEE Sens. J..

[13] Chen D., Varshney P.K. (2007). A survey of void handling techniques for geographic routing in wireless networks. IEEE Commun. Surv. Tutor..

[14] Yang J., Fei Z. ITGR: Intermediate target based geographic routing. Proceedings of the 19th International Conference on Computer Communications and Networks (ICCCN).

[15] Yang J., Fei Z. HDAR: Hole detection and adaptive geographic routing for ad hoc networks. Proceedings of the 19th International Conference on Computer Communications and Networks (ICCCN).

[16] Gabriel K.R., Sokal R.R. (1969). A new statistical approach to geographic variation analysis. Syst. Biol..

[17] Toussaint G.T. (1980). The relative neighbourhood graph of a finite planar set. Pattern Recognit..

[18] Karp B., Kung H.-T. GPSR: Greedy perimeter stateless routing for wireless networks. Proceedings of the 6th Annual International Conference on Mobile Computing and Networking.

[19] Datta S., Stojmenovic I., Wu J. (2002). Internal node and shortcut based routing with guaranteed delivery in wireless networks. Cluster Comput..

[20] Bose P., Morin P., Stojmenovic I., Urrutia J. (2001). Routing with guaranteed delivery in ad hoc wireless networks. Wirel. Netw..

[21] Kranakis E., Singh H., Urrutia J. Compass routing on geometric networks. Proceedings of the 11th Canadian Conference on Computational Geometry.

[22] Kuhn F., Wattenhofer R., Zhang Y., Zollinger A. Geometric ad-hoc routing: Of theory and practice. Proceedings of the Twenty-Second Annual Symposium on Principles of Distributed Computing.

[23] Kuhn F., Wattenhofer R., Zollinger A. Worst-case optimal and average-case efficient geometric ad-hoc routing. Proceedings of the 4th ACM International Symposium on Mobile ad hoc Networking & Computing.

[24] Leong B., Mitra S., Liskov B. Path vector face routing: Geographic routing with local face information. Proceedings of the ICNP 2005 Network Protocols.

[25] Lin C.-H., Yuan S.-A., Chiu S.-W., Tsai M.-J. (2010). Progressface: An algorithm to improve routing efficiency of gpsr-like routing protocols in wireless ad hoc networks. IEEE Trans. Comput..

[26] Clouser T., Miyashita M., Nesterenko M. (2012). Concurrent face traversal for efficient geometric routing. J. Parallel Distrib. Comput..

[27] Deng J., Han Y.S., Chen P.-N., Varshney P.K. Optimum transmission range for wireless ad hoc networks. Proceedings of the 2004 IEEE Wireless Communications and Networking Conference.

[28] Bovy C., Mertodimedjo H., Hooghiemstra G., Uijterwaal H., Van Mieghem P. Analysis of end-to-end delay measurements in Internet. Proceedings of the Passive and Active Measurement Workshop-PAM.

[29] Scalable Network Technologies, Qualnet. http://www.scalable-networks.com.

